# VSI on GMNIA validation in computational structural engineering guest editorial

**DOI:** 10.1016/j.dib.2024.110555

**Published:** 2024-05-31

**Authors:** Adam Jan Sadowski, Mhd Anwar Orabi, Alfredo Camara

**Affiliations:** a Imperial College London, UK; b The University of Queensland, Brisbane, Australia; c Universidad Politécnica de Madrid, Spain

This Virtual Special Issue (VSI) was organised on the eve of the arrival of the new generation of Eurocode 3 where the role of finite element analysis has achieved notable prominence in structural steel design through the widespread adoption and development of GMNIA methods. The aim of this VSI has been to collect in one place an assortment of high quality computational engineering benchmarks for the purposes of model validation (against test data) and / or verification (against an established analytical result). Each publication links to a data repository where empirical and numerical data are paired together wherever both exist, with the text giving the steps necessary for reproduction of the simulation. Approximately 8 GB of data (uncompressed) have been made available across 16 submissions, and we hope that many researchers will find something to aid them in their modelling adventures in this numerical treasure trove. One could take almost any article in this special issue and use it to validate a model knowing that its data is reproducible by numerical simulation as the provided numerical benchmark shows.

Many of the submissions relate to the computational modelling of structural steel. These include data articles on the large-displacement response of moment frames made from high-strength I-sections under combined sway and transverse point loading, on the local buckling response of cold-formed trapezoidal sheets in bending, on the buckling of cold-formed C-shaped columns in compression, on the buckling of thick cold-formed circular hollow sections under three-point bending, on the buckling of rectangular and square hollow sections under *M*-*N* interaction, and on the bearing behaviour of bolted connections. Three submissions released empirical and numerical data pairs for steel components manufactured using wire arc additive manufacturing (WAAM), an emerging technology that places particular demands on the measurement instrumentation which must capture both the complex ‘printed’ geometry and associated displacement or strain fields, as well as on the subsequent modelling protocols that must consume large amounts of continuous information to process these into usable finite element models. These submissions include datasets on the carbon steel bars under tension, sinusoidally-stiffened stub rectangular hollow sections and angle section stub columns, with each component being WAAM-manufactured.

We are very happy to have received submissions on structural materials other than steel: one submission released a large parametric dataset of FRP-strengthened reinforced concrete beams under standard fire exposure, another released data on axially-loaded steel tubes filled with rubberised alkali-activated concrete, and another on the hysteretic response of a ferrocement wall under cyclic loading. Colleagues from aerospace engineering have also provided a dataset on the buckling of composite cylindrical shells under axial compression. It is remarkable that most of the laboratory test programmes now routinely deploy advanced optical reality capture technologies for continuous-field data gathering, including high-resolution laser scanning and Digital Image Correlation (DIC).

To emphasise the universality of a ‘validation benchmark’ in computational engineering, we have permitted ourselves to stretch the VSI's original scope slightly and invite contributions from geotechnical engineering and computational fluid dynamics (CFD). There are therefore submissions offering data to validate codes implementing the Discrete Element Method (DEM) against analytical results and already-established simulation data, triaxial test data of Areolas da Estefânia soil from Lisbon, Portugal, for the validation of constitutive models in soil mechanics simulations, and finally numerical data for the verification of CFD Large Eddy Simulation turbulence models. These three submissions highlight that structural engineers working with steel are blessed (and possibly spoilt!) by a uniquely forgiving material that lends itself uniquely well to a clean empirical and theoretical study. Experiments can be costly but quantities of interest are typically well-defined and measurable to within narrow bounds, which in turn leads to good agreements with numerical models. Other computational engineering disciplines are perhaps not as lucky in this regard, and the fact that controlled experiments are either difficult or impossible to perform does not reduce those disciplines’ need to validate models and software. In such cases what constitutes the ‘ground truth’ may be quite different, and computational tools must be benchmarked or validated in other ways.

The Editors of this Virtual Special Issue are particularly grateful to all participating Authors who dedicated their time, expertise and data towards preparing their submissions, to the Reviewers for providing timely and rigorous reviews, and Data in Brief's own Scientific Editors Dennis Lentferink and Sven Kotowski for their friendly patience, advice and support.

